# Effect of Two Different Training Interventions on Cycling Performance in Mountain Bike Cross-Country Olympic Athletes

**DOI:** 10.3390/sports10040053

**Published:** 2022-04-01

**Authors:** Patrick Schneeweiss, Philipp Schellhorn, Daniel Haigis, Andreas Michael Niess, Peter Martus, Inga Krauss

**Affiliations:** 1 Medical Clinic, Department of Sports Medicine, University of Tübingen, 72076 Tübingen, Germany; philipp.schellhorn@med.uni-tuebingen.de (P.S.); daniel.haigis@med.uni-tuebingen.de (D.H.); andreas.niess@med.uni-tuebingen.de (A.M.N.); inga.krauss@med.uni-tuebingen.de (I.K.); 2 Interfaculty Research Institute for Sports and Physical Activity, University of Tübingen, 72074 Tübingen, Germany; 3 Institute for Clinical Epidemiology and Applied Biometry, University of Tübingen, 72076 Tübingen, Germany; peter.martus@med.uni-tuebingen.de

**Keywords:** polrarized training, off-road cycling, XCO, competition

## Abstract

To improve performance in endurance sports, it is important to include both high-intensity and low-intensity training, but there is neither a universally accepted practice nor clear scientific evidence that allows reliable statements about the predominance of a specific training method. This randomized controlled trial compared the effects of a polarized training model (POL) to a low-intensity training model (LIT) on physiological parameters and mountain bike cross-country Olympic (XCO) race performance in eighteen competitive XCO athletes (17.9 ± 3.6 years). The superiority of one of the two methods could not be shown in this study. The results did not show statistically significant differences between POL and LIT, as both interventions led to slight improvements. However, a small tendency toward better effects for POL was seen for cycling power output during the race (4.4% vs. –2.2%), at the 4 mmol/L (6.1% vs. 2.8%) and individual anaerobic lactate threshold (5.1% vs. 2.3%), and for maximal aerobic performance (4.4% vs. 2.6%), but not for maximal efforts lasting 10 to 300 s. Despite the lack of significant superiority in this and some other studies, many athletes and coaches prefer POL because it produces at least equivalent effects and requires less training time.

## 1. Introduction

Mountain bike cross-country Olympic (XCO) races are denoted as endurance competitions. However, due to their large number of alternating climbs and descents, XCO races are regarded as high-intensive intermittent activities [1,2,3,4]. Elite athletes have to finish 4–7 laps on undulating circuits with technical descents, forest roads, rocky paths and obstacles, which are 4–6 km long, leading to race durations from 80 up to 100 min [5]. Former studies show that XCO races are performed at an average heart rate close to 90% of the maximum, or 84% of maximum oxygen uptake (VO_2_max), and more than 80% of race time is spent above the lactate threshold [2]. The physiological characteristics of XCO athletes indicate that aerobic capacity and the ability to maintain high work rates over a long period are important requirements for competing at a high level. The alternating intensive loads are thereby far above the limit of aerobic endurance performance and thus lead to a rapid reduction in glycogen and an exponential increase in lactate concentration [4], which inhibits aerobic fat metabolism even in phases of low intensity [6].

XCO athletes and other endurance athletes use a variety of strategies to enhance performance, as different training principles related to exercise intensity and volume are known to improve the energy status of the working muscle, resulting in an increased ability to maintain higher muscle power output over time. As evaluated by Coffey and Hawley, there seem to be at least four primary factors that can increase mitochondrial mass and glucose transport capacity in skeletal muscle after various types of exercise training [7]. Two of these primary factors are increases in calcium concentration and changes in energy status in the muscle. A sustained increase in intramuscular calcium, occurring during prolonged endurance training or high training volumes, activates the calcium calmodulin kinases as a mitochondrial biogenesis messenger [7]. The second factor is the relationship between high-intensity exercise and a reduction in ATP concentration in the muscle, leading to a relatively large simultaneous increase in adenosine monophosphate (AMP) that activates the AMP-activated protein kinase (AMPK). Both factors have similar subsequent targets in the skeletal muscle that support the development of the aerobic muscle phenotype [8]. This implies that high mitochondrial oxidative capacity, improved fat oxidation, and increased glucose transport capacity in the skeletal muscle of endurance athletes can be achieved either by high training volumes, high intensities, or various combinations of both [8]. These two signaling pathways show, for example, how different types of endurance training may cause similar adaptive responses.

Training intensity distribution (TID) is an essential tool to prescribe the training stimulus, which depends on the volume and intensity of a single training session but also on a longer-lasting period of training. To enable this differentiation, training intensity is usually divided into different zones based on parameters such as power or speed, heart rate, perceived exertion or other parameters [9]. The most common methods of endurance training in cycling are high-volume, low-intensity training (LIT), lactate threshold training (THR), low-volume, high-intensity (interval) training (HIT), polarized training (POL) and a pyramidal training intensity distribution (PYR) [10,11], although these are not the only methods of TID [9]. Both high-intensity (short-duration) and low-intensity (high-volume) training are important components of a training program, especially for athletes with an intensive competition load. LIT is usually described as exercise performed below the first ventilatory threshold. THR intensity refers to exercise performed between the first and second ventilatory thresholds, and HIT refers to exercise performed above the second ventilatory threshold [12]. The POL concept consists of combined training at low and high intensities, or a gradual reduction of the training volume from LIT to THR and HIT in a pyramid shape [10]. Some studies show that LIT has a positive effect on performance, even if the intensity is much lower than in corresponding competitions [13,14,15]. LIT sessions lead to profound adaptations in skeletal muscle and supporting systems, including increases in the mitochondrial content and respiratory capacity of muscles. Due to the increase in mitochondria, exercise at the same intensity results in a disturbance in homeostasis that is smaller in trained than in untrained muscles. This leads to the assumption that the influence of LIT on already-trained muscles could be limited [16]. A short-term period of HIT is known to improve performance in intense exercise events [16]. However, an athlete’s ability to maintain HIT training sessions is limited [17]. A well-established method to allow more HIT is high-intensity interval training (HIIT), also called transition training, which is defined as an alternation of repeated bouts of high-intensity exercise with recovery periods of low-intensity exercise or complete rest [18]. LIT training sessions seem to be just as important. Even well-trained elite endurance athletes perform a large number of LIT training sessions, despite competing at much higher intensities [12,13]. Seiler and Kjerland estimated that elite endurance athletes perform about 75% of their training volume at intensities below the first ventilatory threshold [12], as this type of training enhances their ability to recover from high-intensity exercises [19]. Briefly summarised, previous studies show that low-intensity and high-volume training, as well as high-intensity and low-volume training, are effective methods to increase athletes’ performance [8,10,13,15,20,21]. These and some other studies indicate that it is important to include both methods in the training programs of athletes who participate in high-intensity sports.

For elite athletes competing in intense endurance competitions (e.g., XCO races), a polarized approach has been suggested as the best distribution of training intensity, with ~75% of the total training volume performed at low intensities, ~15% performed at very high intensities, and the remaining proportions performed somewhere in between [8,12,13,19,20,21]. Nevertheless, there is neither a training concept that is generally accepted in practice nor scientific evidence that allows reliable statements about the superiority of a certain TID method.

To improve training and allow the precise control of respective training sessions, athletes’ specific physiological capabilities should be assessed in appropriate performance tests. For this reason, in recent years, some more comprehensive approaches using both aerobic and anaerobic testing have included several different variables in performance analysis [3,22,23,24,25,26,27,28,29]. In the meantime, performance assessment and training control are increasingly carried out via mobile power meters. These allow more precise training by directly measuring cycling power output and thus have clear advantages over training control via heart rate, since an athlete’s heart rate is an indirect measure of physical strain, which responds with a remarkable delay, particularly during short-term high-intensity exercise, and is influenced by many external factors [30]. The applied system of power training zones developed by Allen and Coggan uses seven power-based training zones (Z1–Z7) instead of the heart rate to represent the full range of physiological responses and to describe the different types of training required to meet the demands of competitive cycling [30].

Due to the aforementioned physiological demands of XCO racing, the present study compared the effects of a polarized training intensity distribution model and a low-intensity training model on physiological performance parameters and race performance in young competitive XCO athletes, whereby training was consistently controlled by cycling power output.

## 2. Materials and Methods

To compare interventional effects on physiological performance parameters and race performance in young competitive XCO athletes, a randomized controlled study with two training groups (POL and LIT) was conducted during the off-season. In the weeks prior to the intervention, athletes trained rather less and predominantly in the aerobic endurance zone, in accordance with their annual periodization.

### 2.1. Participants

**Figure 1:**
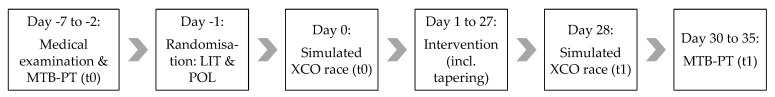
Study flow chart. MTB-PT = mountain-bike-specific performance test; LIT = low-intensity training; POL = polarized training; XCO = mountain bike cross-country Olympic; t0 = baseline test; t1 = retest.

**Figure 2:**
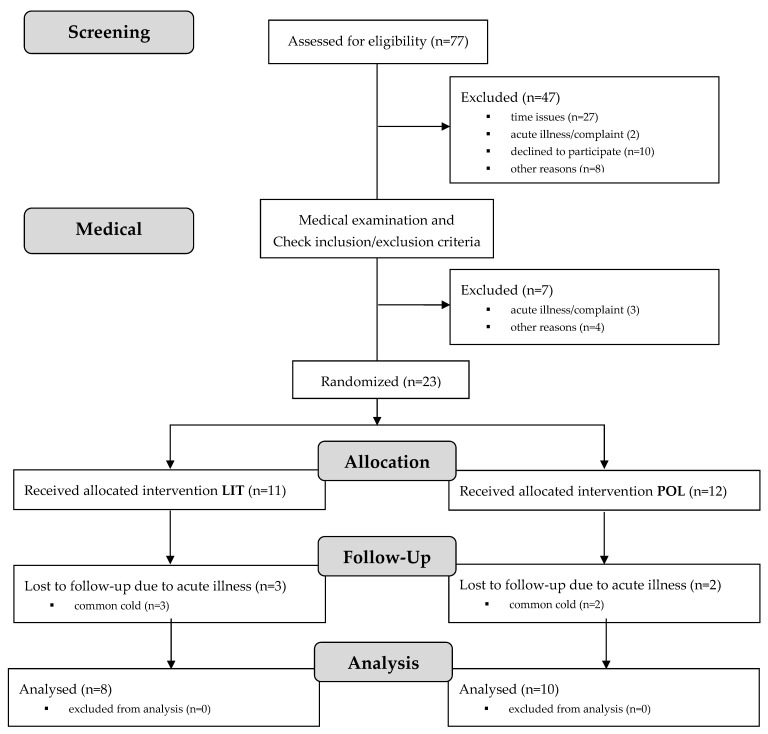
Participant flow diagram. LIT = low-intensity training; POL = polarized training.

Ethical approval was received from the local ethics committee (number 472/2016BO1). The study met the current ethical standards [31] and was registered in a national study registry (number PR020160800134). Eligible participants were recruited through trainers, local cycling clubs, and personal contacts within the mountainbike (MTB) community. After a first telephone screening, 30 athletes were invited for an initial visit. All participants and their legal representative, if athletes were underage, signed an informed written consent form and were screened by a medical doctor for contraindications to the study. After the screening, 23 athletes who started in the current season in the national junior classes U17, U19, or U23 (athletes under 17/19/23 years of age) or the national elite class (older than 23 years) were included in the study. Subsequently to the medical examination and anthropometric measures, all participants performed a mountain-bike-specific performance test (MTB-PT) and a subsequent simulated XCO race within one week (t0). After random block allocation using sex and race (race_1: female athletes and male athletes younger than 17 years; race_2: male athletes 17 years and older), one group performed 3 weeks of polarized training (POL) while the other group continued their regular aerobic endurance training at low intensity (LIT). Following a tapering phase of about one week with significantly reduced training loads, the simulated XCO race was repeated, followed by a post-intervention MTB-PT (t1; Figure 1 and Figure 2).

### 2.2. Procedures

#### 2.2.1. Laboratory Performance Test

The MTB-PT was performed on a Cyclus2 ergometer (RBM elektronik-automation, Leipzig, Germany). A standard MTB frame with an integrated SRM training system (Schoberer Rad Messtechnik, Welldorf, Germany) was fitted to individual requirements (seat post, stem slope, handlebar, and pedal kit). As the gear ratio could be selected individually by electronically simulated shifting, the athletes were able to pedal with their own freely chosen cadence in a seated or standing position. To ensure that the athletes exerted themselves to the maximum, the testing instructor motivated them verbally as much as possible. The SRM training system is considered the gold standard for mobile power meters because of its high validity, reliability, and sensitivity [32,33]. It consists of a mobile power meter (instrumented crank) and power control (PC8; data logger and on-board data display). Cycling power output, cadence, and heart rate were continuously recorded by the PC8 via ANT+ at 1 Hz.

The MTB-PT started with a graded exercise test (GXT) at 80 watts with an increase of 40 watts each 3 minutes until subjective exhaustion followed by 7 min active recovery as described below. Subsequently, athletes proceeded with 4 maximal efforts, alternating with periods of incomplete recovery, pedaling at a load of 1.2 watts*kg^−1^ body mass: (i) 10 s all-out sprint (TT_10_); (ii) 30 s all-out sprint (TT_30_); (iii) 60 s maximal effort (TT_60_); and (iv) a 300 s maximal effort (TT_300_). This test protocol in its entirety was validated with a good predictive power of race performance in XCM [26] and XCO [25]. The mean (10, 30, 60, and 300 s) maximum PO for the maximal efforts was automatically calculated using GoldenCheetah (www.goldencheetah.org; version 3.4). During the last 20 s of each stage of the GXT, capillary blood samples (20 µL) were collected and analysed right after each test (Biosen S-Line, EKF, Cardiff, UK). The individual anaerobic threshold (IAT) was defined as a blood lactate concentration of 1.0 mmol*L^−1^ above the lowest lactate-to-power quotient as proposed by Dickhuth and Röcker [34,35]. The 4 mmol/L lactate threshold (LT_4_) was defined as a blood lactate concentration of 4.0 mmol*L^−1^. The maximal aerobic power (MAP) was calculated using the common equation from Kuipers et al. [36]: MAP = W_f_ + (t/180*40), where W_f_ was the last completed workload of the GXT, and t was the time in seconds of the uncompleted workload.

#### 2.2.2. XCO Race

The simulated XCO race on an official slightly modified Union Cycliste Internationale (UCI) XCO race course in Albstadt, Germany, was organized specifically for this study during the off-season. One lap was 2100 m long, with 130 m of ascent. To account for age and gender differences in the given sample, two races were conducted consecutively for female athletes and under-17 male athletes consisting of 4 laps (race_1), and for male athletes over 17 years, consisting of 6 laps (race_2). To avoid disadvantages due to the starting position, the athletes were positioned by the coaches according to their previous race performance in two starting rows and encouraged to finish the race as fast as possible.

During the intervention period and the two races, the original cranks of the athletes’ bikes were replaced by SRM training systems to monitor recommended training loads and to ensure accuracy and comparability between laboratory and field measurements. All athletes were instructed to refrain from strenuous physical activity, alcoholic drinks, and any medication for at least 24 h both before the MTB-PT and the XCO race and to maintain their habitual preparations for the race. Immediately after the races, athletes were asked about race interruptions, such as falls or technical problems. The mean power output, including zero values of the race (POR), was extracted from the data file with GoldenCheetah as outlined above. Data from athletes with severe health complaints before or during the measurements were excluded.

#### 2.2.3. Training Intervention

The training program was developed and monitored by the coaches involved, while the SRM Training System was used to monitor training and record all data for each training session. The specific intensity for the two different types of training was based on the results of the MTB-PT at t0 by calculating the individual load intensities based on PO at LT_4_ and the maximal effort TT_300_. The individual load intensity for LIT and the low-intensity parts of POL was set at 60% of LT_4,_ which corresponds to training zone two (Z2). The training load for the high-intensity bouts of POL (Z5) was set at 115% of TT_300_. The training stress score (TSS) [30], which takes into account the duration and intensity of a training session to estimate the total training load of the training session, was kept similar for both interventions POL and LIT related to the treatment as a whole. In total, the two training groups POL and LIT corresponded to each other in terms of the approximated physical stress (TSS) of the training, as the POL group trained more intensively, while the LIT group had a higher training volume. The younger and female athletes had different training volumes, which were adequately calculated by the coaches for the respective age group and sex. The TSS for the total training period of 3 weeks for the female and younger athletes in the U17 class was 963 for LIT and POL as well. For the U19/U23/Elite classes, the TSS was 1260 for LIT and 1308 for POL, respectively. Once a week, all athletes completed their usual core training.

Table 1 shows an example of a training schedule for the male age groups U19/23 and Elite.

To describe the total TID more commonly, it was additionally quantified based on the three-phase model of Skinner et al. [37], whereby we determined the total time spent in the three intensity zones: zone A (low intensity, blood lactate concentration (BL) < 2.0 mmol/L), zone B (moderate intensity, BL 2.0 -4.0 mmol/L) and zone C (high intensity, BL > 4.0 mmol/L) using the fixed lactate thresholds according to Mader [38]. The LIT group competed 100%/0.0%/0.0% and the POL group 86.6%/0.0%/13.4% of the total training time in zones A, B and C, respectively.

- Polarized training (POL)

POL included 8 high-intensity training sessions dispersed over three training weeks and 6 to 7 training sessions at low intensity (~25 h of training in total), followed by a one-week tapering phase. Each POL session (Z5) started with a 30-minute warm-up phase at Z1/Z2 and ended with a 30-minute cool-down phase at Z1/Z2. According to the polarized TID, two to three high-intensity interval series alternating 30 s at an intensity of 1.15*TT_300_ (anaerobic capacity; Z6/Z7) and 30 s at Z1/Z2 (active recovery/endurance) were completed, each separated by a 10-minute recovery period at Z1/Z2.

- Low-intensity training (LIT)

The LIT group carried out 14 aerobic endurance training sessions at low intensity (Z2; ~40 h of training in total), followed by a one-week tapering phase.

### 2.3. Statistical Analyses

Data were analysed using IBM SPSS Statistics v.25.0 (IBM Corp, 2017). Descriptive results are presented as mean ± standard deviation (SD). The distribution of data was checked using a Shapiro–Wilk test (*p* > 0.05) and a visual inspection of their histograms, normal Q-Q plots, and box plots. The Mann–Whitney U test for independent samples was used to check whether the central tendencies of the intervention groups were different at t0 (exact significantce: 2*(1−tailed significance)). Between-group effects were tested using the Mann–Whitney U test. The Wilcoxon signed-rank test was used to test whether there was a significant change in the respective PO parameter per intervention group (exact significance: 2*(1−tailed significance)). The level of significance was set at α = 0.05 (two-sided). To visualise differences in cycling power output between t0 and t1, mean absolute percentage error (MAPE) was calculated as the ratio of the difference to the measured value ((t1 − t0)/t0)*100).

## 3. Results

### 3.1. Subjects

After the medical screening, 23 athletes were included in the study and randomised into the two intervention groups, LIT (*n* = 11) and POL (*n* = 12) (Figure 2). Due to the consequences of slight colds, five athletes could not complete all measurements at t0 and t1. Finally, 18 data sets were completely analysed. Table 2 shows the characterization of the intervention groups and the overall sample.

The Mann–Whitney U test showed no differences between the intervention groups at baseline regarding competition class (*p* = 0.76), anthropometric data (age: *p* = 0.90, height (*p* = 0.83), body mass (*p* = 0.46), sex (*p* = 0.90), and physiological variables (IAT: *p* = 0.32, LT_4_: *p* = 0.32, MAP: *p* = 0.52, TT_10_: *p* = 0.63, TT_30_: *p* = 0.83, TT_60_: *p* = 0.36, TT_300_: *p* = 0.27, POR: *p* = 0.27).

### 3.2. MTB-PT and XCO Race

During the race at t0, the track was almost dry. There was neither rain nor any relevant wind, and the average air temperature was about 14 °C. The track conditions at t1 were not as good as at t0; it was much colder (~5 °C), and the track was quite slippery, especially on the wet and partly leaf-covered trials. The mean race duration for female athletes and under-17 male athletes (race_1) was 42.8 ± 4.6 min at t0 and 44.6 ± 6.5 min at t1, respectively. For male athletes over 17 years (race_2), the mean race duration for t0 and t1 was 54.2 ± 1.9 min and 54.6 ± 2.9 min, respectively, which is approximately the recommended national race duration for juniors (50–70 min).

The only variables which indicated negative changes from t0 to t1 were TT_30_ in the POL group and POR in the LIT group, while all others indicated improvements.

**Figure 3:**
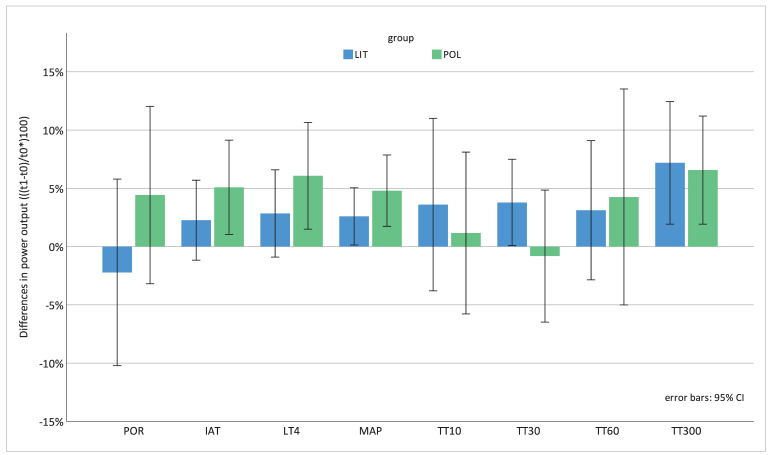
Changes in cycling power output: normalised differences in power output (t1 − t0) (watts in %). Differences are expressed as a percentage of the respective measurement value ((t1 − t0)/t0)*100); error bars: 95% CI; LIT = low-intensity training; POL = polarized training; POR: mean power output during the race; IAT: individual anaerobic threshold; LT4: 4 mmol lactate threshold; MAP: maximal aerobic power; TT_10–300_: time trials (sprint/maximal effort) lasting 10 to 300 s.

As data were not normally distributed, between-group effects were tested using the Mann–Whitney U test. Results did not demonstrate any statistically significant differences between POL and LIT (POR = 0.24, IAT = 0.57, LT_4_ = 0.36, MAP = 0.32, TT_10_ = 0.57, TT_30_ = 0.17, TT_60_ = 0.97, TT_300_ = 0.63). The Wilcoxon signed-rank test showed some significant changes in power output for the POL group, while for the LIT group, only TT_300_ changed significantly from t0 to t1 (Table 3 and Figure 3).

## 4. Discussion

This study aimed to compare two different power-based training interventions on physiological performance parameters and race performance in young competitive XCO athletes. The main findings were (1) no statistically significant differences in the efficiency of the training programs LIT and POL, and (2) a small tendency towards a higher effect of POL compared to LIT.

### 4.1. MTB-PT and XCO Race

The external conditions during the laboratory test were identical for t0 and t1. Even though some athletes experienced the intervals of the MTB-PT as “very hard” (especially TT_30_), all considered the test protocol to be very useful because of its practical relevance. It is remarkable that POL was not able to increase performance during the sprint intervals TT_10_ (+1.2%; *p* = 0.799) and TT_30_ (−0.8%; *p* = 0.721), although 30 s intensive intervals were an important part of the POL group’s training program. The intervention may, therefore, not sufficiently distinguish between the intensity levels. Other measures, such as POR, IAT, LT_4_ and MAP, indicated a small but consistent trend towards a higher effect of POL compared to LIT. This is further confirmed by the results of the Wilcoxon signed-rank test, which showed significant changes in power output for IAT, LT_4_, MAP and TT_300_ for the POL group but only for TT_300_ for the LIT group. However, it must be noted that with *n* = 18 participants, effect sizes of 1.66 would have been needed to achieve a power of 80%.

Unfortunately, the conditions for the races were considerably worse at t1. This could have led to a lower race performance at t1 since the athletes were unable to achieve their full physiological performance due to the technically more challenging conditions caused by the track being partly wet or covered with leaves. Nevertheless, the POL group was able to increase POR from t0 to t1 (+4.4%), while POR decreased in the LIT group (−2.2%). This supports the tendency towards a higher effect of POL, even though this was not statistically demonstrated.

### 4.2. Intervention-Training Program

The training program was designed and monitored by a team of coaches. After the intervention, the recorded training data were screened for the number of exercise units, their duration, and intensity. Both training interventions were feasible and accepted by the athletes. To adjust training individually, load intensities for POL and LIT were based on each athlete’s LT_4_ and TT_300_. Power output at LT_4_ is considered to be similar to the functional threshold power (FTP) in trained cyclists [39]. Nevertheless, functional performance (e.g., FTP) cannot be equated with a physiological parameter (e.g., LT_4_) [39,40]. To ensure comparability, the two training groups corresponded to each other in terms of the approximated physical training stress [30], as the POL group trained more intensively, while the LIT group had a higher training volume. Regarding the distribution of training intensity, it should be noted that the data refer to cycling power output data. The determination of blood lactate concentration might have changed the distribution in the POL group towards a larger proportion of the total training time spent in zone C.

Our results did not demonstrate any statistically significant differences in the efficiency of the training method (POL vs. LIT). This statistically significant difference has also not been demonstrated in some other studies comparing different training intensity distributions such as POL, LIT, THR, PYR or others [41,42], while some other studies indicate that POL leads to greater improvements in endurance performance compared to other training methods. Nevertheless, the small trend towards the superiority of POL is in line with the results of some previous studies that examined the effects of different training modalities on cycling performance [10,13,20,21,43]. Stöggl and Sperlich examined 48 endurance athletes in four intervention groups (LIT, THR, HIIT, POL) before and after a 9-week training period. POL demonstrated the greatest increase in VO_2peak_ (12%), time to exhaustion (+17%), and peak velocity/power (+5% (velocity for running, power for cycling). Velocity/power at 4 mmol·L^−1^ increased after POL (+8%) and HIIT (+5%) [21]. When comparing our results, however, it should be noted that Stöggl and Sperlich examined runners, cyclists, triathletes, and cross-country skiers, and their training intervention lasted nine weeks. Unfortunately, a longer intervention period with precisely defined training programs was not feasible in our case since many athletes and coaches decline to participate in such long interventions at the risk of not achieving the best possible training in the time available. To our knowledge, no comparable data is available for XCO athletes.

In their systematic review and meta-analysis, Rosenblat et al. analysed data from studies that compared the effects of POL vs. THR in endurance athletes. Results showed a moderate effect (ES = −0.66; 95% CI: −1.17 to −0.15), favouring the POL group over the THR group [44]. Neal et al. also showed a slight superiority of POL over THR [43]. They examined 12 male cyclists who completed two 6-week training periods in a randomized crossover design, separated by 4 weeks of detraining. Improvements were greater for POL than THR for PPO (8% vs. 3%; *p* < 0.05), lactate threshold (9% vs.3%; *p* < 0.05), and high-intensity exercise capacity (85% vs. 37%; *p* < 0.05). They concluded that the polarized training distribution results in greater systemic adaptation in already well-trained cyclists. However, these results cannot simply be transferred from THR to LIT.

Inoue et al. compared the effects of HIT and sprint interval training (SIT) on MTB race performance and physiological variables [45]. Similar to our study, the SIT group performed 8–12 30 s supramaximal bouts per session, while the HIT group performed 7 to 10 4–6 min efforts over 6 weeks. Both interventions enhanced simulated MTB performance. Overall, HIT was superior concerning the mean PO in the simulated race (7.8% vs. 5%). Compared to the design of the current study, it should be noted that the race was not a real competition but a simulated laboratory test, and the contents of the intervention differed from those of our study.

### 4.3. Conclusions and Limitations

In this study, both POL and LIT led to slight improvements in performance, and all athletes coped well with the training program, its contents and the training intensities. From the athletes’ point of view, POL was more agreeable and more versatile than LIT. Summarized very briefly, the results of previous studies indicate that POL can lead to a greater improvement in endurance performance than LIT, THR, or other training modalities among well-trained athletes. This tendency applies to our study, although our results could not demonstrate a significant superiority of POL. This may also be due to the short duration of the intervention and the different age groups and thus also the performance levels of the athletes participating in this study. However, implementing such a study in the periodization of professional athletes’ training remains a challenge. Unfortunately, the intervention duration could not be extended due to the organization of training and periodization in competitive cycling.

The diversity of former study designs shows that there are no obvious standards with regard to training modalities or intensity distributions. Training practice in competitive sports indicates that many athletes and coaches favour POL, regardless of scientific evidence. Further studies should therefore try to investigate longer-lasting interventions in larger populations of XCO athletes with a more pronounced POL versus LIT to evaluate the effects of different training intensity distributions and to identify the most suitable form of training for the athletes.

## Figures and Tables

**Table 1:** Race and training schedule for the male age groups U19/23 and Elite.

	t0 (Baseline)	Week_1	f	Week_2	f	Week_3	f	Week_4	f	t_1_ (Retest)
POL	MTB-PT & XCO race	Z5 (1.5 h)	3	Z5 (1.5 h)	3	Z5 (1.5 h)	2	Z5	0	XCO race & MTB-PT
		Z2 (2 h)	1	Z2 (2 h)	2	Z2 (2 h)	3	Z2 (2 h)	1	
LIT	MTB-PT & XCO race	Z5	0	Z5	0	Z5	0	Z5	0	XCO race & MTB-PT
		Z2 (2–3 h)	4	Z2 (2–3.5 h)	4	Z2 (2.5–5 h)	5	Z2 (2 h)	1	

LIT = low-intensity training; POL = polarized training; Z = power-based training zone; f = frequency (per week); h = hours of duration; MTB-PT = mountain-bike-specific performance test (laboratory); XCO race = mountain bike cross-country Olympic race.

**Table 2:** Characterization of the intervention groups and the overall sample.

	Age [years]	Height [m]	Body Mass [kg]	Female [*n*]	Male [*n*]	U17 [*n*]	U19 [*n*]	U23 [*n*]	Elite [*n*]	Race_1 [*n*]	Race_2 [*n*]
LIT (*n* = 8)	17.4 ± 1.9	1.75 ± 0.06	64.2 ± 7.3	2	6	2	4	2	0	3	5
POL (*n* = 10)	18.4 ± 4.7	1.73 ± 0.11	61.2 ± 9.6	2	8	4	4	0	2	5	5
Total (*n* = 18)	17.9 ± 3.6	1.74 ± 0.09	62.5 ± 8.6	4	14	6	8	2	2	8	10

LIT = low-intensity training; POL = polarized training. Values are presented as mean ± standard deviation.

**Table 3:** Race performance and physiological variables: differences in cycling power output (t1 − t0).

		POR	IAT	LT_4_	MAP	TT_10_	TT_30_	TT_60_	TT_300_
LIT (*n* = 8)	Difference	−6.1 ± 25.6 (W) *p* = 0.263	5.9 ± 9.1 (W) *p* = 0.123	8 ± 12.4 (W) *p* = 0.123	8.1 ± 9.8 (W) *p* = 0.161	15.9 ± 73.5 (W) *p* = 0.779	23.1 ± 24 (W) *p* = 0.093	11.4 ± 27.9 (W) *p* = 0.401	20.6 ± 15.8 (W) *p* = 0.025
	MAPE	−2.2 ± 9.6 (%)	2.3 ± 4.1 (%)	2.8 ± 4.5 (%)	2.6 ± 2.9 (%)	3.6 ± 8.9 (%)	3.8 ± 4.4 (%)	3.1 ± 7.1 (%)	7.2 ± 6.3 (%)
POL (*n* = 10)	Difference	11 ± 24.1 (W) *p* = 0.241	10.7 ± 13.3 (W) *p* = 0.028	13.5 ± 14 (W) *p* = 0.022	15.4 ± 15.1 (W) *p* = 0.028	5.4 ± 76.9 (W) *p* = 0.799	−7.8 ± 47.1 (W) *p* = 0.721	13.4 ± 60.2 (W) *p* = 0.333	18.7 ± 21.4 (W) *p* = 0.022
	MAPE	4.4 ± 10.6 (%)	5.1 ± 5.7 (%)	6.1 ± 6.4 (%)	4.8 ± 4.3 (%)	1.2 ± 9.7 (%)	−0.8 ± 7.9 (%)	4.3 ± 12.9 (%)	6.6 ± 6.5 (%)
Total (*n* = 18)	Difference	3.4 ± 25.6 (W)	8.6 ± 11.6 (W)	11.1 ± 13.2 (W)	12.2 ± 13.2 (W)	10.1 ± 73.4 (W)	5.9 ± 40.8 (W)	12.5 ± 47.4 (W)	19.6 ± 18.6 (W)
	MAPE	1.5 ± 10.4 (%)	3.8 ± 5.1 (%)	4.6 ± 5.7 (%)	3.8 ± 3.8 (%)	2.2 ± 9.1 (%)	1.2 ± 6.8 (%)	3.8 ± 10.5 (%)	6.8 ± 6.2 (%)

LIT = low-intensity training; POL = polarized training; POR: mean power output during the race; IAT: individual anaerobic threshold; LT4: 4 mmol lactate threshold; MAP: maximal aerobic power; TT10-300: time trials (sprint/maximal effort) lasting 10 to 300 s; (W) = watts; *p* = two-tailed *p*-value determined by the Wilcoxon signed-rank test; MAPE = mean absolute percentage error (difference expressed as a mean percentage of the respective measurement value ((t1 − t0)/t0)*100).

## Data Availability

The data presented in this study are available on request from the corresponding author.
